# Quantitative Phosphoproteome Analysis Unveils LAT as a Modulator of CD3ζ and ZAP-70 Tyrosine Phosphorylation

**DOI:** 10.1371/journal.pone.0077423

**Published:** 2013-10-30

**Authors:** Mogjiborahman Salek, Simon McGowan, David C. Trudgian, Omer Dushek, Ben de Wet, Georgios Efstathiou, Oreste Acuto

**Affiliations:** 1 T cell Signaling Laboratory, Sir William Dunn School of Pathology, University of Oxford, Oxford, United Kingdom; 2 Central Proteomics Facility, Sir William Dunn School of Pathology, University of Oxford, Oxford, United Kingdom; 3 Molecular Immunology Group, Sir William Dunn School of Pathology, University of Oxford, Oxford, United Kingdom; 4 Computational Biology Research Group, Nuffield Department of Medicine, University of Oxford, Headington, Oxford, United Kingdom; Hungarian Academy of Sciences, Hungary

## Abstract

Signaling through the T cell receptor (TCR) initiates adaptive immunity and its perturbation may results in autoimmunity. The plasma membrane scaffolding protein LAT acts as a central organizer of the TCR signaling machinery to activate many functional pathways. LAT-deficient mice develop an autoimmune syndrome but the mechanism of this pathology is unknown. In this work we have compared global dynamics of TCR signaling by MS-based quantitative phosphoproteomics in LAT-sufficient and LAT-defective Jurkat T cells. Surprisingly, we found that many TCR-induced phosphorylation events persist in the absence of LAT, despite ERK and PLCγ1 phosphorylation being repressed. Most importantly, the absence of LAT resulted in augmented and persistent tyrosine phosphorylation of CD3ζ and ZAP70. This indicates that LAT signaling hub is also implicated in negative feedback signals to modulate upstream phosphorylation events. Phosphorylation kinetics data resulting from this investigation is documented in a database (phosphoTCR) accessible online. The MS data have been deposited to the ProteomeXchange with identifier PXD000341.

## Introduction

The T cell receptor (TCR) plays a central role in adoptive immunity through signaling processes that dictate T cell fate during development and upon exposure to antigen. Deregulation of the underlying signaling circuits may cause immundeficiency and autoimmune disorders. Signals triggered by TCR ligation with peptide-MHC complex are translated into intracellular phosphorylation events by Src and Syk family protein tyrosine kinases (PTK) LCK and ZAP70, respectively. Tyrosine phosphorylation of LAT (The Linker for Activation of T cell) allows the recruitment of signaling protein complexes that activate all major signaling pathways, thus regulating T cell functions [1].

LAT is indispensable for T cell maturation during thymus development [2]. Point mutation of LAT at Y136, the PLCγ-1 binding site, causes massive lymphoproliferation and autoimmunity in mice [3], [4]. These same disorders are also caused by conditional LAT-deletion or expression of LAT mutated at Y136 in peripheral T cells of mice. While LAT-signalosome triggers mostly forward positive signaling, it has been hypothesized that immune disorders could be unleashed by the removal of negative feedback mechanism in TCR signaling associated to LAT [5] rather than altered T cell development in the thymus. However, how LAT may soothe T cell activation and the identity of the targeted signaling components in such a negative feedback are unknown. Various negative regulators through interaction with LAT-signalosome may target upstream signal-triggering modules. A possible negative regulator could be STS1 (Suppressor of T-cell receptor signaling 1) through its interaction with CBL to modulate phosphorylation of ZAP70 [6], [7]. Other possibilities are PTPN6/SHIP1 that targets early signaling modules (TCR-CD3, ZAP70 and LCK) and PTPN7 that is associated with the immunological synapse but its targets remain unknown and in both cases their recruitment to the TCR signalosome is not well defined [1], [8].

Mass spectrometry (MS)-based quantitative phosphoproteomics is a powerful approach to decipher functional signaling networks [9], [10]. Therefore, to further understand the role of LAT in TCR signaling, particularly its possible implication in negative regulations and to identify its targeted molecules, we compared global dynamics of TCR-dependent phosphorylation in normal and LAT-depleted cell lines using the SILAC technology [11], [12]. This allowed us to build TCR signaling networks and investigated its topological distortion in the absence of LAT so as to identify LAT-sensitive and LAT-independent signaling hubs. Our data revealed that TCR-induced transient tyrosine phosphorylation of CD3ζ (Y111) and of ZAP70 (Y492/3) became persistent in the absence of LAT, thus identifying precisely TCR-proximal signaling components that are targeted by a LAT-dependent negative regulatory function. A possible player in this function could be PTPN7. Lack of this negative feedback may explain the dramatic effect on mature T cell homeostasis and suggests the requirement for modulating time and intensity of the exceptionally sensitive TCR signaling machinery [1].

## Methods

**Figure 1:**
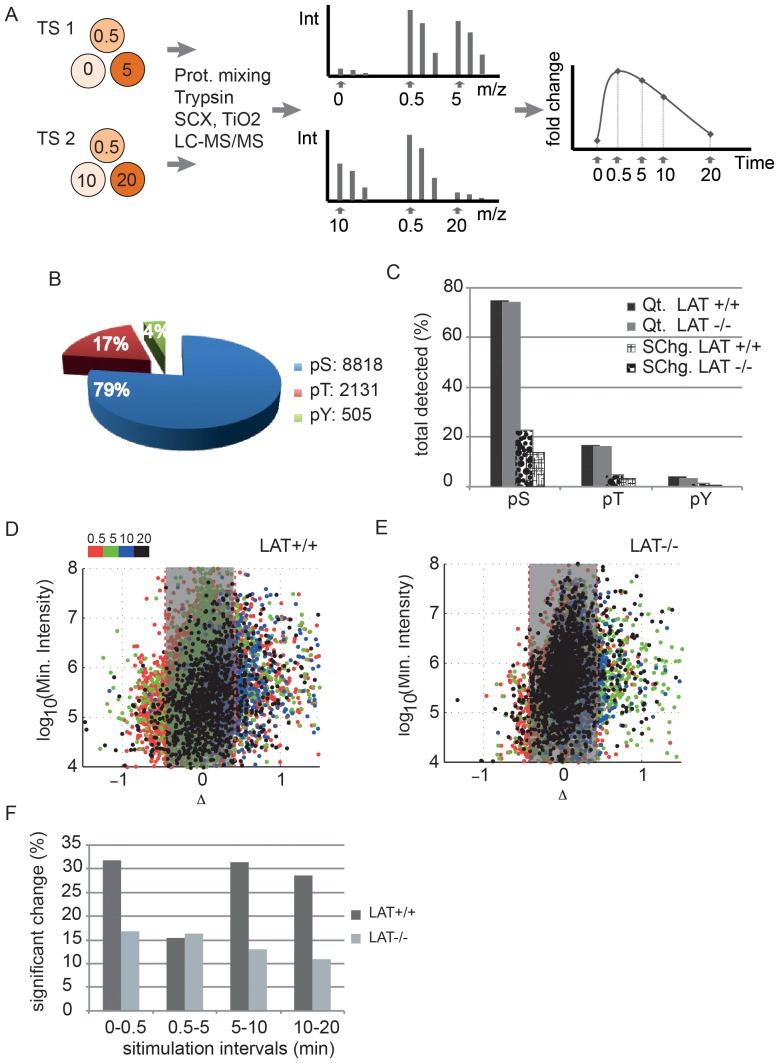
Total phosphorylation in the absence of LAT. (**A**) Phosphoproteomics workflow used in this study starts with labeling Jurkat cell lines in different SILAC media in two time series (TS1 and TS2) as indicated, each composed of three sets of differentially labeled cells. In each TS, cell are activated for the indicated time points and the total protein lysates are equally mixed prior to its digestion by trypsin. The resulted peptides are submitted to strong cation exchange chromatography (SCX) and titanium oxide (TiO2) affinity enrichment prior to liquid chromatography and mass spectrometry (LC-MS/MS) analysis. The data is then analyzed (**see Methods**) and the two time series are normalized using the common time point (0.5 min) so as to obtain a curve representing fold change versus activation time points. (**B**) Pie chart showing the distribution of the total 11,454 unique phosphorylation sites as phospho (p)-Serine (pS), p-Threonine (pT) and p-Tyrosine (pY). (**C**) Bar chart presenting the quantified (Qt) and significant change (SChg.) phosphorylated residues as the percent of the total detected sites in CL20 Jurkat cell lines (LAT+/+) or JCaM2.5 (LAT−/−). (**D, E**) Scatter plot of change in phosphorylation between consecutive time points (Red: 0 to 0.5 min, Green: 0.5 to 5 min, Blue: 0.5 to 10 min, Black: 10 to 20 min) versus the intensity of phosphopeptides - identified in both intact and perturbed cell lines (common peptides) - over each time interval. The grey zone delimits the 95% confidence interval (at ±0.42). (**F**) Bar chart quantifies the common phosphopeptides classified as significant in (**E**) as the percent of the total common peptides over the indicated time intervals.

Main experimental designs are schematically depicted in Fig. 1A and Fig. S1.

### SILAC Labeling

Custom-made RPMI 1640 medium lacking L-Arginine and L-Lysine (Thermo Scientific) was supplemented with 10% dialyzed FCS (Gibco) and either L-Arginine (R^0^) and L-Lysine (K^0^) (CK Gas Products Ltd.), or L-Arginine-^13^C_6_^14^N_4_ (R^6^) (Cambridge Isotope Laboratories, Inc.) and L-Lysine-^12^C_6_^14^N_2_ (4,4,5,5)-^2^H_4_ (K^4^) (Isotec) or L-Arginine-^13^C_6_^15^N_4_- (R^10^) and L-Lysine-^13^C_6_^15^N_2_ (K^8^) (Cambridge Isotope Laboratories, Inc.) at a final concentration of 0.29 mM L-Arginine and 0.219 mM L-Lysine and filter sterilized (0.22 µm pore size, Millipore). Jurkat cells were grown at 37°C in a humidified 5% CO_2_-containing atmosphere for 5–7 cell doublings times in labeling media A, B or C, containing either light: R^0^, K^0^ (A) or medium: R^4^, K^6^ (B) or heavy: R^8^, K^10^ (C).

### Jurkat Cell Lines Stimulation

Two sets of 1×10^8^ cells of each of the three labeling A, B, C were washed twice with serum-free RPMI 1640 and re-suspended each in four tubes of serum-free medium at a concentration of 10^8^ cells/ml. For each time point, cells in four tubes were stimulated for the indicated times using 5 µg anti-CD3 mAb UCHT-1 at 37°C then diluted immediately with chilled PBS, centrifuged at 1200 g for 0.5 min. The supernatant was discarded and the cell pellet lysed for 10 min with ice-cold lysis buffer (20 mM Tris pH7.5, 150 mM NaCl, 1% Dodecyl-β-D-maltoside (Calbiochem), 1 mM Na_3_VO_4_, protease inhibitor cocktail (Roche), phosphatase inhibitor cocktail 1 and 2 (Sigma)). Lysates were cleared by centrifugation at 14,000×g for 5 min and the four samples of each time point were pooled. For each time series (TS1∶0 min (A), 0.5 min (B), 5 min (C) and TS2∶10 (A), 0.5 (B), 20 (C) min), the corresponding time points lysates were mixed in a 1∶1∶1 ratio of protein content (measured by the NanoDrop® ND-1000 UV-Vis Spectrophotometer). The two stimulation series had one common and two varying time points, resulting overall in five time points per experiment (0, 0.5, 5, 10 and 20 min). Stimulation efficiency of each individual sample was determined by western blot using anti-phosphotyrosine (pY) (4G10, Millipore; PY99, Santa Cruz Biotechnology). JCaM2.5 cells and JCaM2.5 cells stably transfected with LAT were stimulated with 10 µg/ml CD3 antibody (UCHT1) for varying times, cells lysed, and lysates subjected to total ZAP70 and ZAP-pY493 (Santa Cruz Biotech. Inc.) immunoblot.

### Protein Precipitation and Digestion

Total protein in the two time series were precipitated overnight in 0.1 M ammonium acetate in chilled methanol. The next day, samples were centrifuged at 6000 g for 30 min and washed 3 times with chilled methanol and 2 times with chilled acetone. Total Protein pellet was re-suspended in 8 M urea, 15 mM Tris pH 8. Protein mixture was reduced in 10 mM DTT (final concentration), 30 min 37°C and then alkylated with 50 mM IAA at 37°C in the dark.

After dilution with 15 mM Tris, pH 8, to 2 M urea, trypsin was added in 1/100 enzyme/protein ratio and the samples were incubated overnight at 37°C.

### SCX and TiO2 Enrichment

Tryptic peptide mixture was acidified prior to separation by strong cation exchange (SCX) chromatography as described elsewhere [13]. In brief, acidified peptides were loaded onto PolySULFOETHYL A column (100×9.5; 5 µm; 200-Å), equilibrated with buffer A (7 mM KH2PO4, pH 2.65, 30% ACN (vol/vol)), on an ÄKTA Purifier chromatography system (Amersham Biosciences). Peptides were eluted by applying a 35 min gradient, from 0 to 25% buffer B (7 mM KH2PO4, 350 mM KCl, pH 2.65, 30% ACN (vol/vol)). Column was washed with 100% (B) and re-equilibrated with (A). SCX fractions were collected in a range spanning the flow through to early wash with 100% B.

Titanium oxide enrichment was performed as described in details elsewhere [14]. Minor modifications were related to the chemical reagent (glutamic acid (GA) instead 2,5-of dihydroxybenzoic acid (DHB)) to reduce nonspecific binding of acidic peptides and the enrichment in batch as follows. Each fraction was diluted 4/10 with a saturated solution of GA in 80% ACN/3%TFA. 10 µl of TiO2 stationary phase (10 ug in 100 ul) - washed 1 time in 80% ACN/0.1% TFA and 1 time in 0.2% NH3 water in 40% ACN (pH≥10.5), re-equilibrated in 80% ACN/0.1% TFA - was added to each fraction and incubated 1 h at room temperature on rotating wheel. TiO2 beads were then treated as described elsewhere [14]. Peptides were eluted directly into a 96 well plate, acidified by formic acid (FA) (4/50), lyophilized and desalted using OASIS® (HLB µElution plate 30 µm, Waters). Peptides were then re-suspended in 0.1% FA prior to LC-MS/MS measurement.

### Liquid Chromatography and Mass Spectrometry

LTQ-Orbitrap mass spectrometer (ThermoElectron) was coupled online to a nano-LC Ultimate 3000 (Dionex). To prepare the analytical column, C_18_ material (ReproSil-Pur C18-AQ 3 µm; Dr. Maisch GmbH, Germany) was packed into a spray emitter (75 µm ID, 8 µm opening, 70 mm length; New Objectives) using a high-pressure packing device (NanobaumeTM, Western Fluids Engineering). Mobile phase A consisted of 0.1% formic acid in water and B of 80% ACN, and 0.1% formic acid in water.

The 5 most intense peaks of the MS scan were selected for MS2 scans in the ion trap (full MS scan at resolution 60,000 and mass range 400–1,600 maximum filling of 1×10^6^ ions for maximum injection time of 500 ms. For MS2 scans, maximum filling was 1×10^4^ ions with maximum injection time of 150 ms. Minimum signal was 1000 counts and normalized collision energy set to 35 with activation time of 30 ms. Dynamic exclusion was set to 60 seconds, repeat count 1, exclusion mass width relative to low and high was 20 ppm, expiration count and S/N threshold were 2 and 3.0, respectively, and multistage activation was enabled with neutral loss of phosphoric acid from 2–5 charge states.

### Mass Spectrometry Data Processing

Raw data files were processed using an early version of MaxQuant software [15]. In the first step, peak lists files (.msm files) for SILAC medium- and heavy-labeled peptide as well as unassigned labeling state were generated using the Quant.exe. These files were submitted to Mascot (version 2.3.01) search engine using Ensemble Human (GRCh37.59), in house generated, target decoy database. Trypsin specific cleavage, except if the cleavage site is followed (at the C-terminal) by proline, was set and maximum of three miss-cleavages were allowed. N-terminal protein acetylation, oxidation of methionine, phosphorylation of serine, threonine and trypsin were set as variable and Carbamidomethyl cysteine was set as fixed modification. For peak list files corresponding to medium- or heavy-labeled peptides, additional fixed modification were Arg^6^ and Lys^4^ or Arg^10^ and Lys^8^, respectively. Peak list of the remaining unassigned SILAC state were searched with all the above-labeled modifications as variable. Three labeled amino acids per peptide were allowed.

In the second step, Identify.exe is using the identification files (.dat) from Mascot search engine in combination to raw, and protein sequence data files to perform identification, quantitation, integration of the results, statistical validation and estimation of FDR (set to 0.01%) and finally to write results in various output files (.txt). For phosphopeptides, the site assignment was scored by MaxQuant (for more details see [15]).

Complete time series for phosphorylation sites were generating by merging the two sub-series using custom perl scripts. Briefly, phosphorylated peptide sequences were retrieved from the MaxQuant ‘modifiedPeptides.txt’ output file. For each phosphorylation site among these sequences the best supporting spectrum match (highest mascot score) was retrieved from the MaxQuant ‘evidence.txt’ file. SILAC ratios for the time-points in each sub-series were combined by normalizing to the common 0.5 min time point, present as the medium labeled sample (B). Phospho-peptides and sites were re-mapped to the complete Ensembl Human protein sequence database, to ensure complete representation of ambiguity in the site-to-protein mapping.

### Protein Interaction Networks

To generate protein-protein interaction networks a custom perl script was used to map identified phospho-sites to interaction data from STRING-DB. For the set of all phospho-sites, protein mappings were assembled and reduced to gene-level identifiers. Interactions between the set of phospho-proteins with a STRING interaction score >0.7 were retrieved from STRING-DB. Topology of the phospho-protein networks was examined using custom Perl scripts. Random sampling of subset networks from both the phospho-proteins identified in this study and the set of all STRING interactions was performed. Distributions of edge/node number and network degree were obtained. Cytoscape2.8.1 software [16] was used to visualize these networks.

### PhosphoTCR Database

A MySQL database was created to store the analysis results and a set of custom Perl-cgi scripts written to enable interaction with the data via a web interface. All MS/MS spectra used to generate the peptide data within the database are available as static images (from Central Proteomics Facilities Pipeline: CPFP [17]) via links within the interface. The database is available at: https://cellline.molbiol.ox.ac.uk/phos/cgi-bin/PhosphoTCR.cgi.

### MS Raw Data

The mass spectrometry proteomics data have been deposited to the ProteomeXchange Consortium (http://proteomecentral.proteomexchange.org) via the PRIDE partner repository [18] with the dataset identifier PXD000341.

## Results

### LAT-dependent TCR-induced Cellular Phosphorylations

To determine the role of LAT in regulating TCR-induced phosphoproteome dynamics, we labeled Jurkat (CL20) and its LAT-deficient variant (JCam2.5) cell lines using SILAC approach and quantified relative changes in phosphopeptide abundance at 0.5, 5, 10 and 20 min after TCR stimulation (**Fig. 1A** **and Fig. S1**). To determine a threshold above or below which a change in phosphopeptide abundance could be considered statistically significant, we computed a global *P*-value for fold changes from two data sets obtained from cells independently stimulated in identical conditions (**File S1, Figures S4–S6**). This procedure established a threshold of ±0.42 fold change over background with 95% confidence interval (i.e. a |fold change| ≥ 0.42 for a given phosphopeptide is significant with *P*-value = 0.05). In all experiments a total of 11,454 unique phosphorylation sites were identified in 2336 proteins. Phosphorylation kinetics data resulting from this investigation is documented in a database (phosphoTCR) that can be accessed online (**see Methods**). As expected, serine phosphorylation (pS) represents the largest fraction of identified sites (79%) followed by threonine (pT, 17%) and tyrosine (pY, 4%) (**Fig. 1B**). Surprisingly, in spite of the central position of LAT in TCR signaling, under the stimulatory conditions used, global phosphorylation events in the absence of LAT were not affected severely, as illustrated in **Fig. 1C**. Typically, over 70% of the phosphorylation sites detected were quantified in both intact and LAT-deficient Jurkat cell lines. However, in the absence of LAT, 33% less phospho-peptides show significant change upon stimulation **(** **Fig. 1C** **)**. To better appreciate details of significant changes in both cell lines, we analyzed the distribution of the changes in phosphopeptides commonly identified in both cell lines for which data were available over the five time points, as shown in **Fig. 1D and E**. This set of peptides represents the most robust identification and quantitation as they are detected over 4 independent stimulations and two independent MS analyses (TS1 and TS2, see Fig. 1 A). When expressed as the percent significant change of the total fraction in each interval, it shows 40–50% less phosphorylation abundance change in the absence of LAT (**Fig. 1F**) thus allowing a more accurate comparison of LAT-dependent phosphorylation.

### LAT-deletion Selectively Perturbs Well-defined Functional Hubs in TCR Signaling Networks

**Figure 2:**
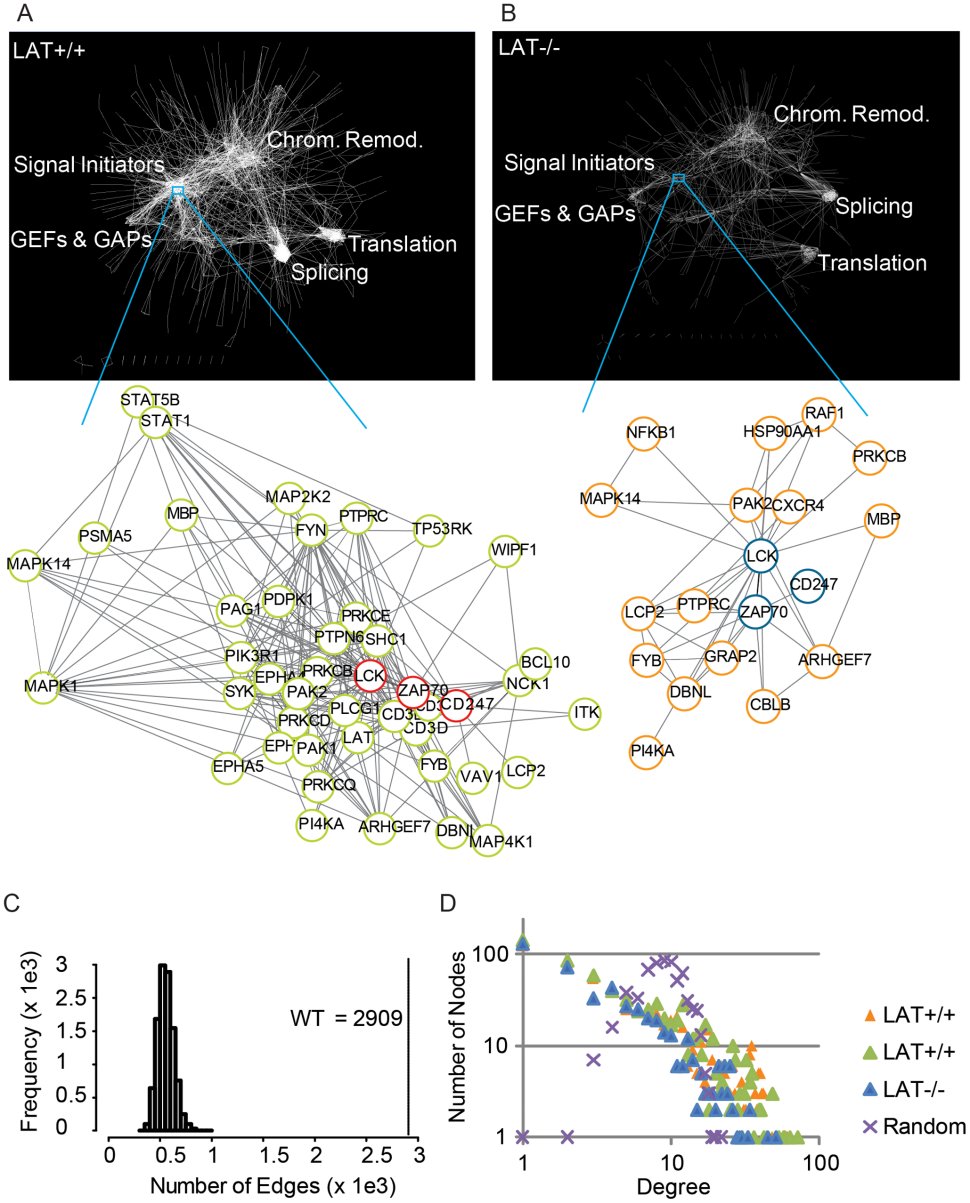
Topology of TCR signaling networks and effects of LAT deficiency. (**A** and **B**) Phosphorylation-specific networks based on integration of our phosphoproteomic data with protein-protein interactions in the STRING database. Note that both networks show constellation of hubs characterized by interacting proteins function. Zooms into the signaling hub of LAT efficient (LAT+/+) or deficient (LAT−/−) cell lines shows first neighbors (pistachio green or orange circles) of CD3ζ, LCK and ZAP-70 (red or blue circles). (**C**) The number of edges and (**D**) degree distribution for experimental and random networks. Orange and pistachio-green triangles correspond to degree distribution in networks based on data from two replicas in Jurkat CL20 cell line. Blue triangles and purple crosses correspond to networks based on the data in LAT-deficient cell line and randomly selected proteins, respectively.

To define the topology of TCR-induced signaling networks and its perturbation in the absence of LAT, we analyzed the phosphoproteomics data in the context of protein-protein interaction (PPI) [19]. Towards this goal, we first built networks based on our data in LAT-efficient and deficient cell lines. We then compared their intrinsic properties to highlight LAT-dependent distortions of the global signaling network (**Fig. 2** **and Methods**). Phosphorylation-specific networks in intact (**Fig. 2A**) and LAT-deficient cell lines (**Fig. 2B**) show constellations of functional hubs. The most affected hub, by the absence of LAT, is composed of signaling proteins as shown in the magnification of the “Signal Initiators” hub. Less perturbed hubs are those indicated as “GEF & GAPS”, “Chromatin Remodeling”, “Splicing” and “Translation”. The latter two are composed of more tightly assembled nodes, probably indicating that they arrange into stable complexes, characteristic of multi-protein machineries.

To assess the specificity of the generated networks we computed the frequency of edges and average neighbors in subset interaction networks, sampled from the experimental data and randomly selected proteins. The number of edges (**Fig. 2C**) (average neighbors, not shown) is significantly lower in the random compared to the phospho-specific network, indicating lower networking in the absence of LAT. As expected for scale free networks, the degree distribution (**Fig. 2D**) of the intact and perturbed networks obey to a Poisson distribution [20], [21] and can therefore be considered phospho-specific. Similarly to previous parameters, the degree distribution is slightly lower in the ‘perturbed’ network, which accounts for globally lower phosphorylation (see **Fig. 1F**) and, consequently, lower networking.

Although LAT deletion does not raze the global architecture of the TCR signaling networks, it reduces the global TCR-induced phosphorylation. In particular, the hub, through which LAT transduces the input signals, seems to disaggregate. This identifies LAT-dependent modules that could account for the onset of LAT-dependent autoimmunity and highlights alternative signaling routes (e.g. the “GEF & GAPS” hub; see the discussion).

### LAT Modulates Phosphorylation of CD3ζ and ZAP70

**Figure 3:**
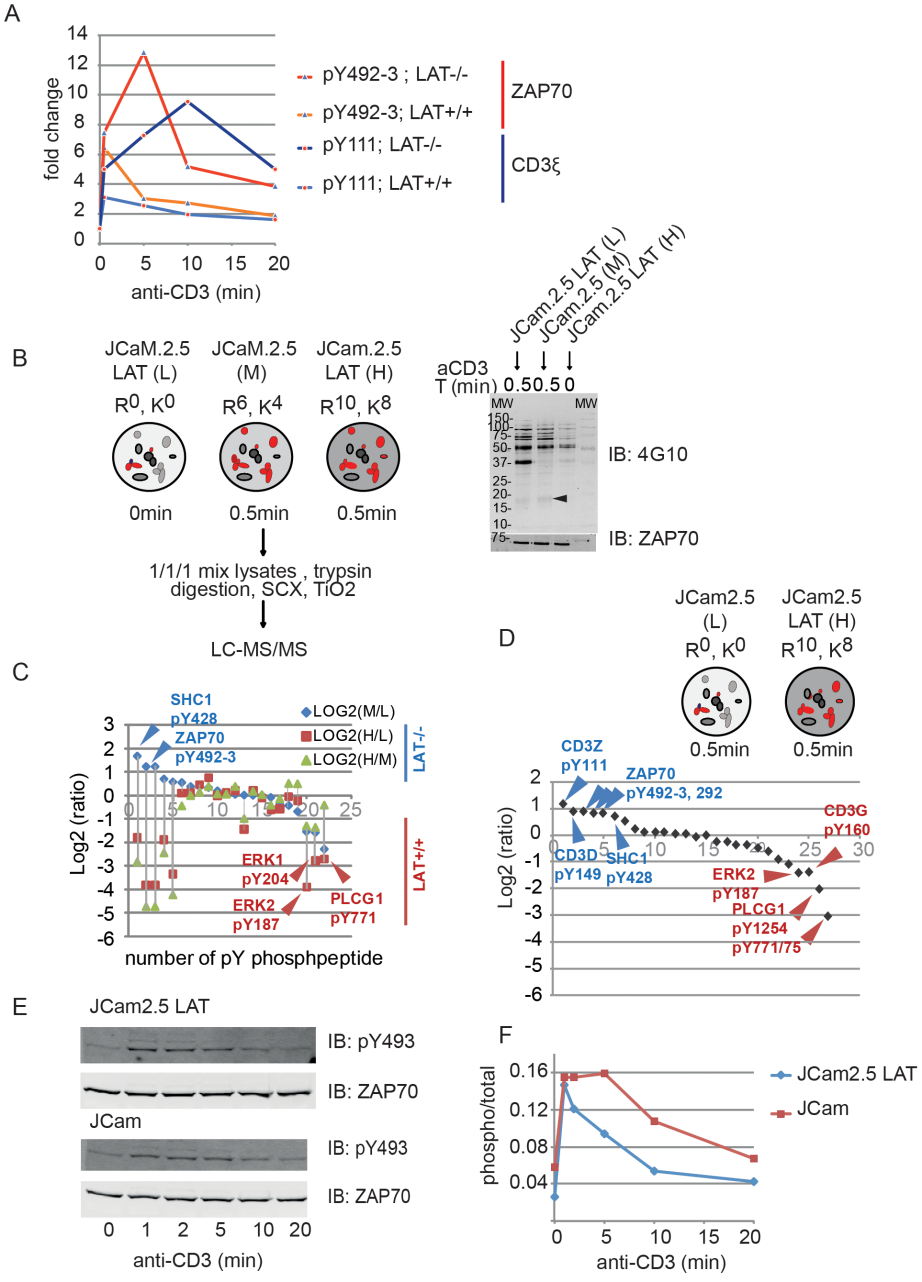
LAT regulates phosphorylation of ZAP70 and CD3Z. (**A**) The comparison of CD3ζ and ZAP70 phosphopeptides kinetics in CL20 and JCaM2.5 cell lines. (**B**) SILAC labeling strategy for direct comparison of peptide-specific phosphorylation in JCaM.2.5 and JCam2.5-LAT cell lines. Cell lines grown in SILAC media (Arginine: R and Lysine: K) were activated (or not) for the indicated time points. Total protein extract were equally mixed and subjected to phosphoproteomics analysis (for details see **Methods and Fig. 1S**). Anti-pY detection of the cell lysates was used to control the activation. Anti-ZAP70 antibodies were used to control the loading. The arrow at 18 KDa shows a band that probably corresponds to CD3ζ. L, M and H stand for Light, Medium and Heavy amino acids combinations. (**C**) S-shape graphic showing log2-transformed ratios for tyrosine phosphorylated peptides signals in different experimental conditions: M/L (Blue diamonds; peptide signal from 0.5 min activated JCam2.5/resting JCam2.5LAT), H/L (light-red squares; JCaM2.5LAT activated/JCaM2.5 resting), H/M (Pistachio-green triangles; JCaM2.5LAT activated/JCaM2.5 activated). (**D**) Similar to **C** except that only activated cell lines were confronted, as indicated. The graphic shows log2-transformed H/L ratio (black diamonds; JCaM2.5LAT activated/JCaM2.5 activated). (**E**) LAT-dependent phosphorylation of ZAP70 was tested as indicated. (**F**) Quantitation of the immunoblots in (**E**). This file contains: additional information on the analysis of global dynamics of TCR-induced phosphorylation; methods to evaluate experimental error and define activation threshold; supporting references.

After examining the global aspects of our data (**Fig. S2**), a closer inspection of peptide-specific phosphorylation shows that kinetics of the pY492-93 (ZAP70) and pY111 (CD3ζ) last longer in the absence of LAT (**Fig. 3A**). In order to confirm the LAT dependency of ZAP70 and CD3ζ phosphorylation, we SILAC labeled JCam2.5 and JCam2.5-LAT Jurkat cell lines to quantify differences by directly confronting the two cell lines (**Fig. 3B**). We followed an identical workflow as previously (**Fig. S1**) except we now compare directly two cell lines for a single activation time point (0.5min). As previously reported for mice CD4+ T cells freshly deprived of LAT [5], total tyrosine phosphorylation patterns (**Fig. 3B** **,** anti-pY immunoblot) in tested Jurkat cell lines are similar except LAT (absent in JCam.2.5) and some differences in the intensity of a few protein bands. In particular a band at around 18 KDa, corresponding to the expected molecular weight of CD3ζ, is of higher intensity in the absence of LAT (**Fig. 3B**). The MS results are presented as S-shaped curve showing log2-transformed ratios of peptide-specific intensity in tested cell lines (**Fig. 3C**). In order to increase the sensitivity of the analysis and further strengthen these results we reduced the number of labels in a new experiment, as increasing the number of isotopic labels increases the number of MS signals and proportionally reduces the sensitivity. Therefore, the new experiment consisted in confronting the activated LAT-deficient and efficient cell lines, without the zero time point (**Fig. 3D**). MS results show that tyrosine phosphorylation is consistently lower for ERK1/2 (pY204, pY187) and PLγC1 (pY1254, pY771/775) and higher for ZAP70 (pY492-93) and CD3ζ (pY111) in the absence of LAT. To further support LAT-dependent kinetics of ZAP70 phosphorylation, untransfected JCaM2.5 and stably LAT transfected JCaM2.5 were stimulated for varying times and lysates subjected to total ZAP70 and ZAP-pY493 immunoblot (**Fig. 3E, 3F** **and Fig. S3**). These results corroborate the MS data (**Fig. 3A**) that ZAP70 phosphorylation last longer in the absence of LAT.

Taking together our data expose the increased phosphorylation of CD3ζ and ZAP-70 in the absence of LAT. Importantly it reinforces the notion that LAT has a negative regulatory role on upstream signaling events that could contribute to the underlying molecular mechanism at the basis of pathological conditions associated with LAT deletion.

## Discussion

We deployed quantitative phosphoproteomics on Jurkat cell lines to better understand the dynamics and the intricate interplay of signaling components in TCR-regulated phosphoproteome networks and its distortions induced by LAT-deletion. In particular we intended to identify defective signaling nodes that may cause the onset of the LAT signaling pathology (LSP) and possibly shed light on its underlying molecular mechanisms.

Previous studies demonstrated that LSP is characterized by hyper-proliferation of freshly LAT-deprived peripheral CD4+ T cells and therefore LAT mutations causing partial loss of function might not be due to thymic selection altering TCR affinity threshold but rather to peripheral T cell malfunction [5]. Although the onset of the disorder is dependent on an initial engagement of TCR, CD4+ cells that undergo hyper-proliferation express extremely low levels of TCR and thereby become unresponsive to stimuli. In agreement with our observation in Jurkat cell lines, tyrosine phosphorylation patterns of wild type and freshly LAT-deprived activated CD4+ T cells were similar [5].

However, here we provide quantitative data allowing more accurate comparison. Our data show that in the absence of LAT, protein phosphorylation persists not only on tyrosine but also serine and threonine and that some TCR-triggered signals are transduced independent of LAT.

From a global perspective, LAT deletion decreases the overall level of phosphorylation and consequently interferes with phosphorylation-specific protein networking. However less stout, the outline of the signaling networks architecture in the absence of LAT remains comparable to the intact network. These results suggest that despite the disaggregation of LAT-dependent diversification module, some signals still propagate. One such group of proteins that contain GEF-GAP domains is forming a distinct network that seems unaffected in the absence of LAT.

In agreement with previous studies, we show that LAT deficient cell lines are defective in ERK (Ras) and PLCγ1 (phosphatidylinositol) pathways [22], [23]. LAT-independent signals could possibly be conveyed via SLP-76 and/or GIT-PIX-PAK pathways recruited to the membrane through integrin signaling constituents. Indeed, previous reports have shown that SLP-76 seems to be phosphorylated in the absence of LAT and that GIT-PIX-PAK complex could be recruited to the plasma membrane at the integrin activation site through GIT interaction with Paxillin [24], [25]. Although in this study we have tested time points outside the phosphorylation peak of GIT and PIX, at around 2 min [10], nevertheless we observed that in the absence of LAT, phosphorylation sites of GTI-1 and PIX (Arhgef-6), in resting and activated cells (e.g. 0.5 and 5 min) remained comparable (see the kinetics in the phosphoTCR database).

Upstream of LAT, we found that the tyrosine phosphorylation of CD3ζ and ZAP70 was persistent in the absence of LAT. It seems therefore that unchecked phosphorylation of CD3ζ leads to further recruitment of ZAP-70 and its phosphorylation in turn escapes negative regulation.

Different molecular mechanisms are reported to modulate activation of TCR and ZAP70 through dephosphorylation (PTPN22 and SHP1) and/or ubiquitin-induced degradation of signaling molecules (STS1/2) [1]. Our data indicates that a possible phosphatase that could modulate TCR and ZAP70 phosphorylation may be PTPN7. It is the unique phosphatase for which we observe a differential phosphorylation in the absence of LAT (**Fig. S3E**). Its phosphorylation on S143 [26] increases upon TCR triggering in the presence of LAT while it remains unchanged in its absence. S143 is within the phosphatase domain (aa 97 to 350) and therefore could impact PTPN7 phosphatase activity.

In conclusion our data indicates that: (1) TCR triggering induces phosphorylation-specific signaling networks organized in well-defined functional hubs, (2) perturbation of TCR singling by LAT deletion does not alter the global architecture of this network, (3) however, while modules through which LAT transduces the input signals seems to disaggregate, (4), signals still propagate to downstream components. (5) One such hub is formed by GEF-GAP domain containing proteins such as those involved in GTI-PIX-PAK pathway. (6) Unexpectedly, in perturbed modules, tyrosine phosphorylation of CD3ζ and ZAP70 last longer in the absence of LAT which indicates that LAT, in addition to its role in forward signaling, has negative regulatory function modulating upstream phosphorylation events possibly through phosphatase such as PTPN7. Our data open new avenues to study LAT-independent pathways and to establish further LAT-dependent nature of PTPs negative regulation of the TCR and ZAP-70 phosphorylation.

## Supporting Information

Figure S1 **The workflow to measure kinetics of TCR-induced phosphoproteome.** Jurkat cells were split in three sets, and each one grown in indicated SILAC media (distinct combination of K and R isotopomers, **see Methods**). Here we show details of the workflow only for a single series of time points, as the complementary time series was submitted to the same workflow (**see** **Fig. 1A**). After activation for different times (t1, t2 and t3), cells were lysed and protein extracts were equally mixed prior to the trypsin digestion (**see Methods**). Resulted peptides mixture was submitted to strong cation exchange chromatography (SCX) followed by titanium oxide (TiO2) affinity enrichment of phosphopeptides and then analyzed by LC-MS/MS. The MS data were processed and analyzed using MaxQuant and CPFP. The results are documented in PhosphoTCR database (**see Methods**).
(PDF)

Figure S2 **Dynamics of TCR-induced phosphorylation. (A)** Clusters grouping phosphopeptides with similar kinetic profiles were generated using ProteinCenter software (Proxeon Biosystems A/S, Odense, Denmark). **(B)** Kinases whose phosphorylation peaks at the indicated time points. The color code corresponds to different clusters. **(C and D)** Peptide specific profiles for some known and unexpected proteins in cluster 1 and 2. **(E)** Comparison of PTPN7 pS143 kinetics in LAT-efficient and -deficient cell lines.
(PDF)

Figure S3 **LAT-dependent phosphorylation of ZAP70.** Additional biological replicates for anti-pY293 ZAP70 and anti-ZAP70 immunoblots performed on indicated cell lysates (see also **Fig. 3E and 3F**).
(PDF)

Figure S4 **Workflow for evaluating intra-experimental error and determining an activation p-value.** Three sets of CL20 Jurkat T cells were labeled in culture media with distinct combinations of amino acid isotopomers. The three samples were stimulated using anti-CD3 antibodies for 0 min (*k* = 0), 0.5 min (*k* = 1), and 0.5 min (*k* = 2). The two 0.5 min stimulations are to serve as controls to determine the total intra-experimental error. After digestion of protein lysates with trypsin, the samples were split in two (split sample *i = *1 or *i* = 2) before phosphopeptide enrichment, three LC-MS/MS injections, and data analysis are performed. This workflow produced two phosphoprylation test datasets (one from each split sample) that each included quantitative phosphorylation data on the three stimulation conditions. The red and blue segments delimit experimental steps for which distinct errors were estimated.
(PDF)

Figure S5 **Intra-experimental error in phosphoproteomics experiments.** (**A**) Comparison of the two split samples, which includes error contributions only from steps following sample splitting (i.e. peptide enrichment, LC-MS/MS, and data analysis as delimited by the vertical blue segment in **Fig. S4**). (**B**) Comparison of the two 0.5 min stimulations, which includes error contribution from every step from initial culture (as delimited by the vertical red segment in **Fig. S4**). (**C–D**) Correlation between changes in phosphorylation and absolute intensity: shown are scatter plots of the absolute intensity versus the change in phosphorylation between (**C**) the split samples (as in **A** and in **Fig. S4**, the blue segment) and (**D**) the two 0.5 min stimulations (as in **B** and **Fig. S4**, the red segment).
(PDF)

Figure S6 **Experimental error in independent biological replicates.** (**A**) The histogram of the differences in the change of phosphorylation (γ = JR2-JR1) in peptides common to two biological replicates (JR1 and JR2). Dashed red lines indicate 95% confidence intervals based on total intra-experimental error (**Fig. S5B**) whilst grey dashed lines indicate 95% confidence intervals [−1.5,1.1] based on inter-experimental error. (**B**) Scatter plots of JR1 vs. JR2 of the two biological replicates (correlation coefficient r = 0.54). We note that the correlation on the log2-transformed data is similar to published studies. Diagonal white line represents a slope of 1.
(PDF)

File S1 **Supporting text.** This file contains: additional information on the analysis of global dynamics of TCR-induced phosphorylation; methods to evaluate experimental error and define activation threshold; supporting references.
(DOC)
