# Supplementary figures and images for: Quantitative Phosphoproteome Analysis Unveils LAT as a Modulator of CD3ζ and ZAP-70 Tyrosine Phosphorylation
Source: PLoS One. 2013 Oct 30;8(10):e77423. doi: 10.1371/journal.pone.0077423 (PMC3813684; doi:10.1371/journal.pone.0077423)

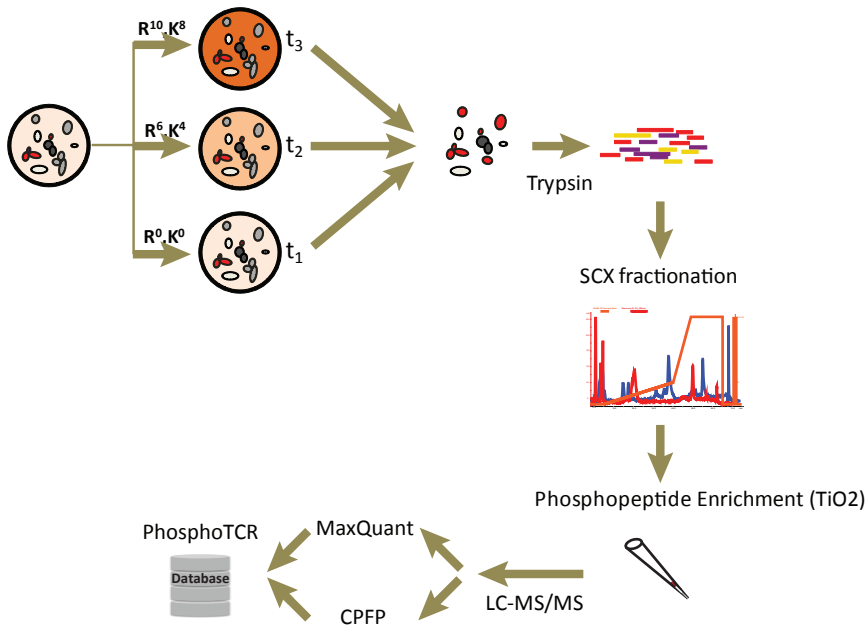

Figure S1

Supplement: Figure S1 — The workflow to measure kinetics of TCR-induced phosphoproteome. Jurkat cells were split in three sets, and each one grown in indicated SILAC media (distinct combination of K and R isotopomers, see Methods). Here we show details of the workflow only for a single series of time points, as the complementary time series was submitted to the same workflow (see Fig. 1A ). After activation for different times (t1, t2 and t3), cells were lysed and protein extracts were equally mixed prior to the trypsin digestion (see Methods). Resulted peptides mixture was submitted to strong cation exchange chromatography (SCX) followed by titanium oxide (TiO2) affinity enrichment of phosphopeptides and then analyzed by LC-MS/MS. The MS data were processed and analyzed using MaxQuant and CPFP. The results are documented in PhosphoTCR database (see Methods). (PDF) [file pone.0077423.s001.pdf]

A

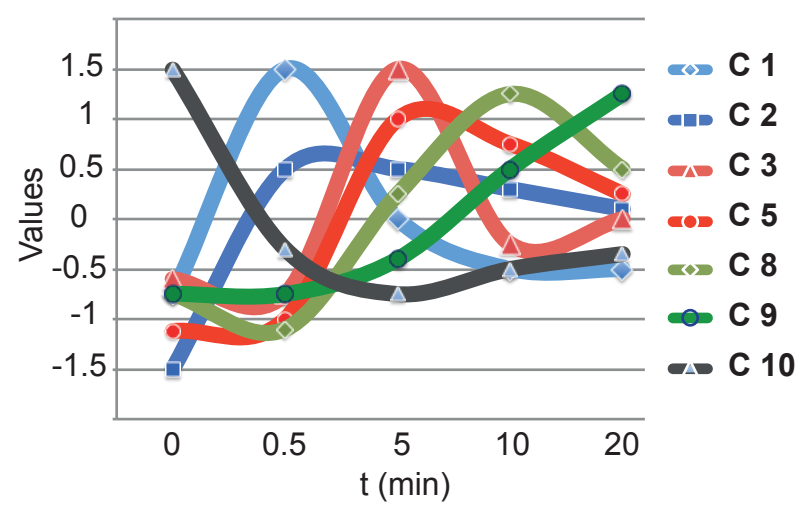

B

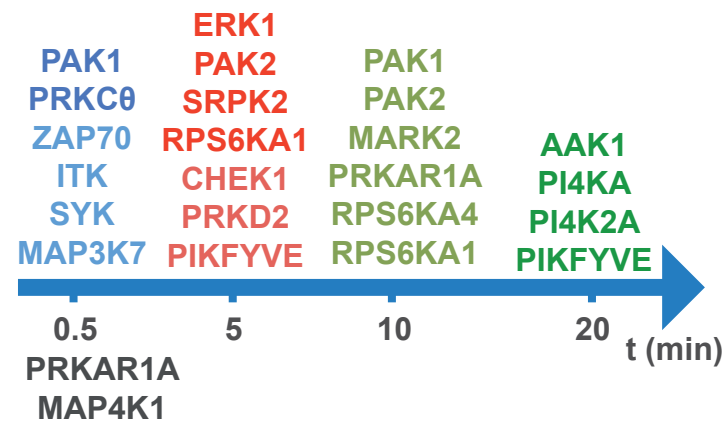

C

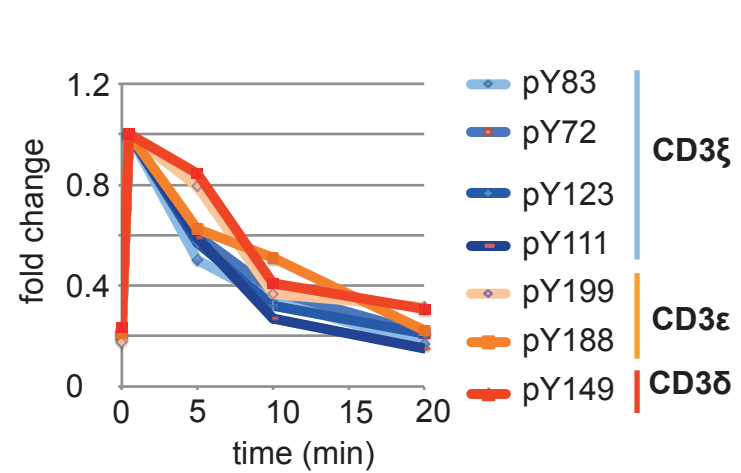

D

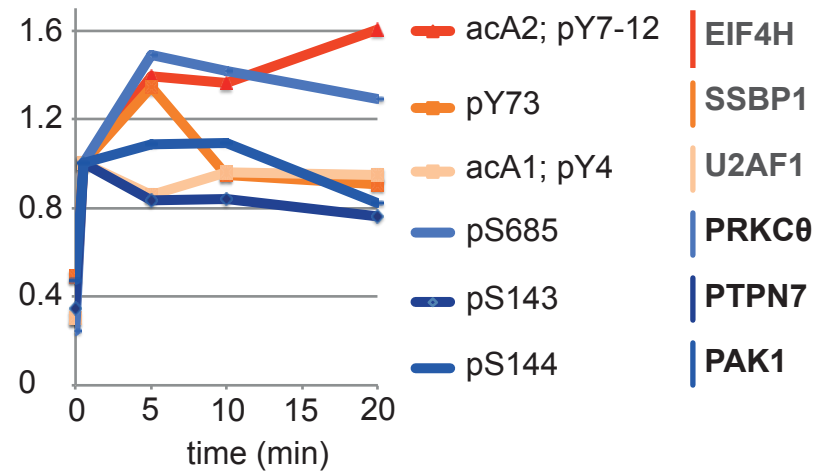

E

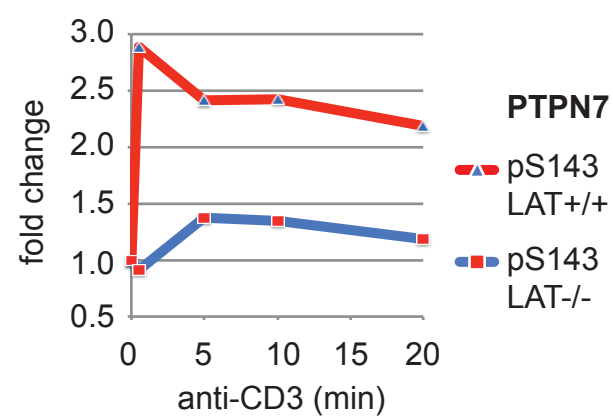

Figure S2

Supplement: Figure S2 — Dynamics of TCR-induced phosphorylation. (A) Clusters grouping phosphopeptides with similar kinetic profiles were generated using ProteinCenter software (Proxeon Biosystems A/S, Odense, Denmark). (B) Kinases whose phosphorylation peaks at the indicated time points. The color code corresponds to different clusters. (C and D) Peptide specific profiles for some known and unexpected proteins in cluster 1 and 2. (E) Comparison of PTPN7 pS143 kinetics in LAT-efficient and -deficient cell lines. (PDF) [file pone.0077423.s002.pdf]

A

JCam2.5 LAT

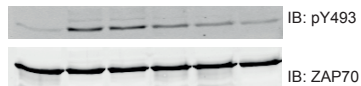

JCam

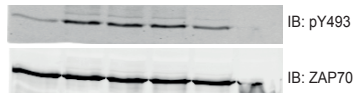

0 1 2 5 10 20

anti-CD3 (min)

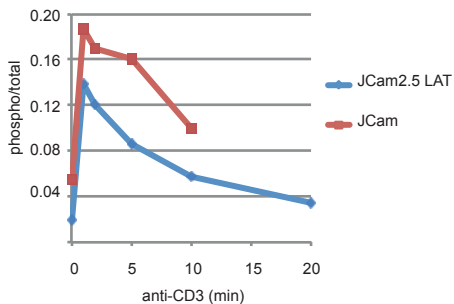

B

JCam2.5 LAT

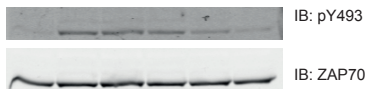

JCam

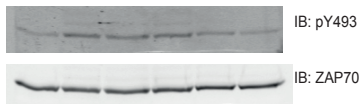

0 1 2 5 10 20

anti-CD3 (min)

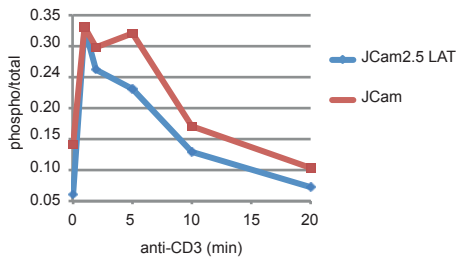

Figure S3

Supplement: Figure S3 — LAT-dependent phosphorylation of ZAP70. Additional biological replicates for anti-pY293 ZAP70 and anti-ZAP70 immunoblots performed on indicated cell lysates (see also Fig. 3E and 3F ). (PDF) [file pone.0077423.s003.pdf]

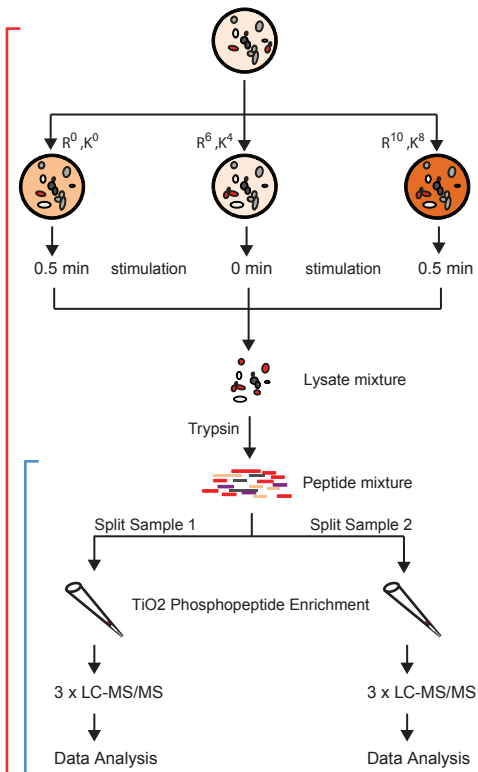

Figure S4

Supplement: Figure S4 — Workflow for evaluating intra-experimental error and determining an activation p-value. Three sets of CL20 Jurkat T cells were labeled in culture media with distinct combinations of amino acid isotopomers. The three samples were stimulated using anti-CD3 antibodies for 0 min (k = 0), 0.5 min (k = 1), and 0.5 min (k = 2). The two 0.5 min stimulations are to serve as controls to determine the total intra-experimental error. After digestion of protein lysates with trypsin, the samples were split in two (split sample i = 1 or i = 2) before phosphopeptide enrichment, three LC-MS/MS injections, and data analysis are performed. This workflow produced two phosphoprylation test datasets (one from each split sample) that each included quantitative phosphorylation data on the three stimulation conditions. The red and blue segments delimit experimental steps for which distinct errors were estimated. (PDF) [file pone.0077423.s004.pdf]

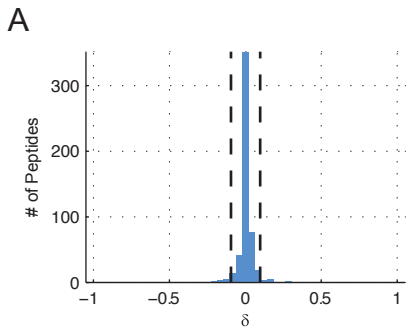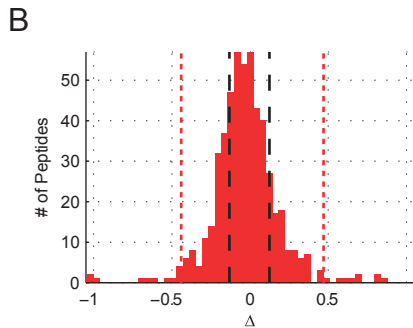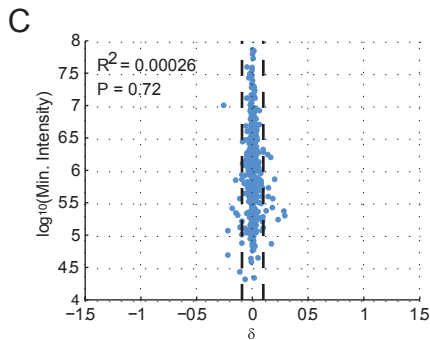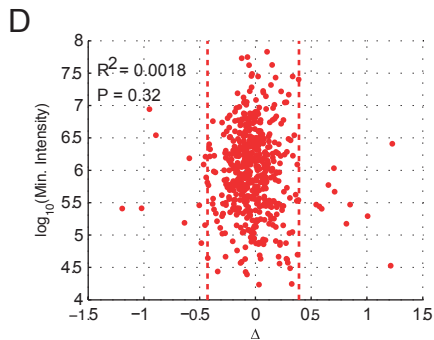

Figure S5

Supplement: Figure S5 — Intra-experimental error in phosphoproteomics experiments. (A) Comparison of the two split samples, which includes error contributions only from steps following sample splitting (i.e. peptide enrichment, LC-MS/MS, and data analysis as delimited by the vertical blue segment in Fig. S4). (B) Comparison of the two 0.5 min stimulations, which includes error contribution from every step from initial culture (as delimited by the vertical red segment in Fig. S4). (C–D) Correlation between changes in phosphorylation and absolute intensity: shown are scatter plots of the absolute intensity versus the change in phosphorylation between (C) the split samples (as in A and in Fig. S4, the blue segment) and (D) the two 0.5 min stimulations (as in B and Fig. S4, the red segment). (PDF) [file pone.0077423.s005.pdf]

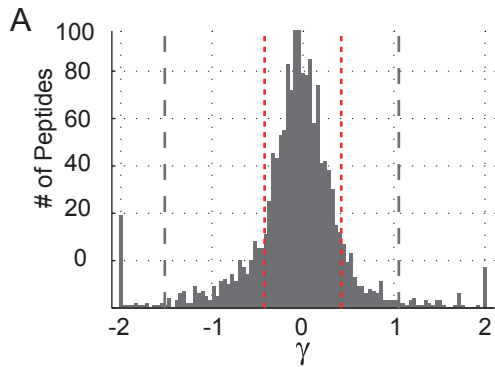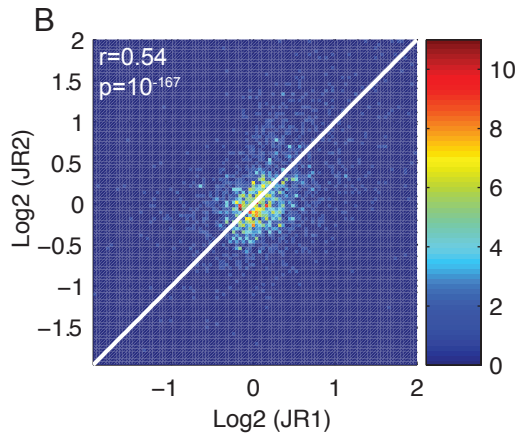

Figure S6

Supplement: Figure S6 — Experimental error in independent biological replicates. (A) The histogram of the differences in the change of phosphorylation (γ = JR2-JR1) in peptides common to two biological replicates (JR1 and JR2). Dashed red lines indicate 95% confidence intervals based on total intra-experimental error (Fig. S5B) whilst grey dashed lines indicate 95% confidence intervals [−1.5,1.1] based on inter-experimental error. (B) Scatter plots of JR1 vs. JR2 of the two biological replicates (correlation coefficient r = 0.54). We note that the correlation on the log2-transformed data is similar to published studies. Diagonal white line represents a slope of 1. (PDF) [file pone.0077423.s006.pdf]
