# Supplementary material for: Quantitative Phosphoproteome Analysis Unveils LAT as a Modulator of CD3ζ and ZAP-70 Tyrosine Phosphorylation
Source: PLoS One. 2013 Oct 30;8(10):e77423. doi: 10.1371/journal.pone.0077423 (PMC3813684; doi:10.1371/journal.pone.0077423)
Supplement: File S1 — Supporting text. This file contains: additional information on the analysis of global dynamics of TCR-induced phosphorylation; methods to evaluate experimental error and define activation threshold; supporting references. (DOC) [file pone.0077423.s007.doc]

**SUPPORTING TEXT FILE S1**

1. **ANALYSIS OF GLOBAL DYNAMICS OF TCR-INDUCED PHOSPHORYLATION**
2. **EVALUATION OF THE EXPERIMENTAL ERROR AND DEFINITION OF THE ACTIVATION THRESHOLD (FIGURES S4 TO S6)**
3. **SUPPORTING REFERENCES**
4. **ANALYSIS OF THE GLOBAL DYNAMICS OF TCR-INDUCED PHOSPHORYLATION**

To grasp the global dynamics of TCR-induced signaling, identified phosphopeptides in CL20 Jurkat cells, were clustered by kinetic-profiles similarity (**Fig. S2A**). Each cluster shows distinct phosphorylation dynamics such as transient (C1, C3), long lasting (C2, C5, C8), increasing at late time point (C9) and early decreasing phosphorylation (C10) behavior in various signaling proteins. For clusters peaking at 0.5 min (C1, C2), some representative proteins are shown in **Fig. S2C** and **S2D**, respectively. This includes the TCR-CD3 subunits (**Fig. S2C**) for which the very early increase in phosphorylation abundance is to be expected; the same was observed for PAK1 involved in the regulation of cytoskeleton dynamics and for PTPN7 and PKC-theta, essential regulators of ERK pathways and the activation of NF-kappa-B and JUN, respectively . Interestingly, proteins involved in RNA translation initiation (EIF4H), splicing (U2AF1; )) and transcription (SSBP1) show increase tyrosine phosphorylation located at the N-terminus and in the single stranded DNA biding site of SSBP1 . Although, further targeted studies are required to clarify the precise role of such early tyrosine phosphorylation, it underlines a possible role of TCR in modulating the flow of information at different stages of gene expression.

The variation in abundance of phosphorylation of some kinases at different time points is shown in **Fig. S2B**. Noticeably, in addition to protein tyrosine kinases (PTKs) ZAP-70, SYK and ITK, changes in phosphorylation in serine/threonine kinases (STKs) such as PAK1, PKC-theta and MAP3K7 were also detected. This is consistent with the activation of actin cytoskeleton rearrangement and the MAPK and NF-κB pathways, respectively, known to be stimulated after TCR ligation ((**Fig. S2C, S2D**). Interestingly, at the initial activation time point (0.5 min) some known negative regulators of the TCR signaling such as MAPK4K1 (HPK1) and PKA (the regulatory subunit PRKAR1A) (**Fig. S2B**) show a decrease in phosphorylation that may suggest that triggering is not only consisting in activating latent molecular mechanisms but may also be removing regulatory constraints.

To explain the longer lasting phosphorylation of pY492-93 in ZAP70 and pY111 in CD3ζ in the absence of LAT (**Fig. 3A**), we searched for possible alterations of phosphorylation patterns in tyrosine phosphatases detected in this study. We found that the only tyrosine phosphatase whose phosphorylation was perturbed in the absence of LAT was PTPN7 (**Fig. S2E**). The phosphorylation abundance of S143 shows an increase of approximately two folds in the LAT-sufficient Jurkat cells while it remains almost unchanged in the absence of LAT.

1. **EVALUATION OF THE EXPERIMENTAL ERROR AND DEFINITION OF THE ACTIVATION THRESHOLD (FIGURES S4 TO S6)**

***Workflow to estimate intra-experimental error***

To quantify intra-experimental errors in our phosphoproteomic data, we designed a workflow (**Fig. S4**) whereby a cell population was cultured in three SILAC labeling conditions (**identical to those in Methods, unless stated otherwise**); two samples of 20 x 10^6^ cells were taken from each condition at 1 x 10^8^ /ml final concentration and stimulated identically (0.5 min stimulation) and one sample served as a reference (0 min stimulation). Importantly, following trypsin digestion, the peptide mixture was split and analyzed in parallel to produce two sets of data (one for each split sample) each containing phosphopeptides from 0, the first 0.5 and second 0.5 min stimulation. Comparison of the first (or the second) 0.5 min stimulation between split sample 1 and 2 provides an estimate of the intra-experimental error associated with all steps that follow and include peptide enrichment **(Fig. S4,** blue segment**)**. The comparison of the two 0.5 min stimulations in split sample 1 or 2 provides an estimate of the total intra-experimental error (**Fig. S4,** red segment).

***Defining p-values for significant fold-changes based on total intra-experimental error estimates***

Let y_i,j,k_ represent the phosphorylation of peptide *j* for split sample *i* (*i* = 1 or *i* = 2) at time point *k* (*k*=0 for 0 min, *k*=1 for the first 0.5 min, and *k*=2 for the second 0.5 min). The data is normalized so that y_i,j,0_ = 1 for all *i* and *j*. With these definitions, we can compute the change in phosphorylation between the two 0.5 min time points as,

∆_i,j_ = y_i,j,2_ – y_i,j,1_ (1)

Note that ∆_i,j_ represents an estimate of the total intra-experimental error. In **Fig. S5B** we show the distribution of ∆_1,j_ (a similar distribution is found for ∆_2,j_ , which is not shown) and in **Table S1** we summarize the distribution statistics.

As expected, the distribution to be approximately normal with a mean near zero and relatively symmetric 95% confidence intervals. Therefore, peptides that exhibit a change in phosphorylation outside of this range are significant at the 5% level.

The mean 95% confidence intervals from the ∆_1,j_ and ∆_2,j_ distributions are [-0.435, 0.430]. Therefore, changes in phosphorylation outside of this range are deemed significant with a P-value of 0.05 (i.e. observing a change in phosphorylation for a specific peptide that is either > 0.430 or < -0.435 is significant with P < 0.05).

As additional controls, we correlate the raw intensity values to ∆_1,j_ (**Fig. S5D**) or ∆_2,j_ (not shown) and find no significant correlations, implying that the data used for evaluating the error are not biased by the absolute intensity values.

***Determining the dominant source of intra-experimental error by comparing peptides from split samples***

To investigate which components of the workflow are dominant contributors to the total intra-experimental error we computed the change in phosphorylation between the two split samples (*i*=1 and *i*=2),

δ_k,j_ = y_2,j,k_ – y_1,j,k_ (2)

Note that now we collapse index *i* and keep index *k*.

The distributions of δ_0,j_ (**Fig. S5A**) and δ_2,j_ (not shown) produce similar confidence intervals (**Table S1**), which are both substantially smaller than the confidence intervals produced by the distributions of ∆_i,j_, the total intra-experimental error (**Fig. S5B** and **Table S1**). It follows that the total intra-experimental error is dominated by errors that arise before enrichment (workflow components that take place before the enrichment of the peptide mixture: see **Fig. S4**). Note that δ_1,j_ = 0 for all *j* because the two independent injections are normalized based on the *k* = 1 time point.

***Inter-experimental error in biological replicas***

To evaluate inter-experimental errors we used two independent biological replicas. Each replica was based on stimulating CL20 Jurkat T cells in two series of 3 time points using anti-CD3, as described in the **Methods** and in . The two stimulation series had one common and two varying time points, resulting overall in five time points per independent experimental replica (0 min, 0.5 min, 5 min, 10 min, and 20 min).

We first identified peptides that were present in both independent replicas (common peptides in replicas JR1 and JR2) and then calculated the difference in the change in phosphorylation (γ = JR2-JR1). In an idealized experiment, the change in phosphorylation would be identical in the two independent experiments and therefore the difference between the two would always produce 0. Instead, we find a broad distribution (**Fig. S6A**). This histogram shows that the 95% confidence intervals based on intra-experimental error (**Fig. S5B,** red dashed lines) are much smaller than the 95% confidence intervals based on inter-experimental errors (grey dashed lines [-1.518,1.054]). This result indicates that inter-experimental errors increase the error margin by more than 2-fold compared to intra-experimental errors.

The usual approach to evaluating inter-experimental errors (biological reproducibility) in changes in phosphorylation of peptides common to two replicas is to examine their correlation . In an idealized experiment, the change in phosphorylation would be identical in the two independent experiments resulting in a perfect correlation. We find (**Fig. S6B**) correlations (r=0.54) that are similar to published values . This is in agreement with the observation that usually for a given phosphorylation its abundance kinetics profile (peaking time, transient or persistent nature etc…) is similar between biological replicas however the absolute intensity is prone to larger variation.

Table S1: Summary Statistics of Experimental Error

| Error | Mean | 95% CI | |
| --- | --- | --- | --- |
| ∆_1,j_ (Fig. S5B) | -0.017 | -0.430 | 0.390 |
| ∆_2,j_ (not shown) | 0.033 | -0.440 | 0.470 |
| δ_0,j_ (Fig. S5A) | 0.005 | -0.094 | 0.098 |
| δ_2,j_ (not shown) | -0.007 | -0.170 | 0.150 |
| γ (Fig. S6A) | -0.0883 | -1.518 | 1.054 |

1. **SUPPORTING REFERENCES**

**1. Sbrissa D, Ikonomov OC, Fu Z, Ijuin T, Gruenberg J, et al. (2007) Core protein machinery for mammalian phosphatidylinositol 3,5-bisphosphate synthesis and turnover that regulates the progression of endosomal transport. Novel Sac phosphatase joins the ArPIKfyve-PIKfyve complex. J Biol Chem 282: 23878-23891.**

**2. Sommer K, Guo B, Pomerantz JL, Bandaranayake AD, Moreno-Garcia ME, et al. (2005) Phosphorylation of the CARMA1 linker controls NF-kappaB activation. Immunity 23: 561-574.**

**3. Isakov N, Altman A (2002) Protein kinase C(theta) in T cell activation. Annu Rev Immunol 20: 761-794.**

**4. Baier-Bitterlich G, Uberall F, Bauer B, Fresser F, Wachter H, et al. (1996) Protein kinase C-theta isoenzyme selective stimulation of the transcription factor complex AP-1 in T lymphocytes. Mol Cell Biol 16: 1842-1850.**

**5. Zuo P, Maniatis T (1996) The splicing factor U2AF35 mediates critical protein-protein interactions in constitutive and enhancer-dependent splicing. Genes Dev 10: 1356-1368.**

**6. Matsuoka S, Ballif BA, Smogorzewska A, McDonald ER, 3rd, Hurov KE, et al. (2007) ATM and ATR substrate analysis reveals extensive protein networks responsive to DNA damage. Science 316: 1160-1166.**

**7. Sells MA, Knaus UG, Bagrodia S, Ambrose DM, Bokoch GM, et al. (1997) Human p21-activated kinase (Pak1) regulates actin organization in mammalian cells. Curr Biol 7: 202-210.**

**8. Brown JL, Stowers L, Baer M, Trejo J, Coughlin S, et al. (1996) Human Ste20 homologue hPAK1 links GTPases to the JNK MAP kinase pathway. Curr Biol 6: 598-605.**

**9. Jin G, Klika A, Callahan M, Faga B, Danzig J, et al. (2004) Identification of a human NF-kappaB-activating protein, TAB3. Proc Natl Acad Sci U S A 101: 2028-2033.**

**10. Shui JW, Boomer JS, Han J, Xu J, Dement GA, et al. (2007) Hematopoietic progenitor kinase 1 negatively regulates T cell receptor signaling and T cell-mediated immune responses. Nat Immunol 8: 84-91.**

**11. Di Bartolo V, Montagne B, Salek M, Jungwirth B, Carrette F, et al. (2007) A novel pathway down-modulating T cell activation involves HPK-1-dependent recruitment of 14-3-3 proteins on SLP-76. J Exp Med 204: 681-691.**

**12. Vang T, Torgersen KM, Sundvold V, Saxena M, Levy FO, et al. (2001) Activation of the COOH-terminal Src kinase (Csk) by cAMP-dependent protein kinase inhibits signaling through the T cell receptor. J Exp Med 193: 497-507.**

**13. Vang T, Abrahamsen H, Myklebust S, Horejsi V, Tasken K (2003) Combined spatial and enzymatic regulation of Csk by cAMP and protein kinase a inhibits T cell receptor signaling. J Biol Chem 278: 17597-17600.**

**14. Brockmeyer C, Paster W, Pepper D, Tan CP, Trudgian DC, et al. (2011) T cell receptor (TCR)-induced tyrosine phosphorylation dynamics identifies THEMIS as a new TCR signalosome component. J Biol Chem 286: 7535-7547.**

**15. Olsen JV, Vermeulen M, Santamaria A, Kumar C, Miller ML, et al. (2010) Quantitative phosphoproteomics reveals widespread full phosphorylation site occupancy during mitosis. Sci Signal 3: ra3.**

**16. Rigbolt KT, Prokhorova TA, Akimov V, Henningsen J, Johansen PT, et al. (2011) System-wide temporal characterization of the proteome and phosphoproteome of human embryonic stem cell differentiation. Sci Signal 4: rs3.**
